# Avian influenza A (H5N1) outbreaks in different poultry farm types in Egypt: the effect of vaccination, closing status and farm size

**DOI:** 10.1186/s12917-018-1519-8

**Published:** 2018-06-18

**Authors:** Jean Artois, Carla Ippoliti, Annamaria Conte, Madhur S. Dhingra, Pastor Alfonso, Abdelgawad El Tahawy, Ahmed Elbestawy, Hany F. Ellakany, Marius Gilbert

**Affiliations:** 1 Spatial Epidemiology Lab. (SpELL), Université Libre de Bruxelles, Brussels, Belgium; 2 Istituto Zooprofilattico Sperimentale dell’Abruzzo e del Molise ‘G. Caporale’, Teramo, Italy; 3 Food and Agriculture Organization of the United Nations, Rome, Italy; 4 National Center for Animal and Plant Health (CENSA), OIE Collaborating Center for Reduction of the Risk of Disasters in Animal Health, 32700 San José de las Lajas, Mayabeque Cuba; 5 Faculty of Veterinary Medicine, Damanhour University, Damanhour, Egypt; 6 Fonds National de la Recherche Scientifique, Brussels, Belgium

**Keywords:** Avian influenza A (H5N1), Egypt, Poultry farms, Risk or protective factors, Biosecurity

## Abstract

**Background:**

The Avian Influenza A (H5N1) virus is endemic in poultry in Egypt. The winter of 2014/2015 was particularly worrying as new clusters of HPAI A (H5N1) virus emerged, leading to an important number of AI A (H5N1) outbreaks in poultry farms and sporadic human cases. To date, few studies have investigated the distribution of HPAI A (H5N1) outbreaks in Egypt in relation to protective / risk factors at the farm level, a gap we intend to fill. The aim of the study was to analyse passive surveillance data that were based on observation of sudden and high mortality of poultry or drop in duck or chicken egg production, as a basis to better understand and discuss the risk of HPAI A (H5N1) presence at the farm level in large parts of the Nile Delta.

**Results:**

The probability of HPAI A (H5N1) presence was associated with several characteristics of the farms. Vaccination status, absence of windows/openings in the farm and the number of birds per cycle of production were found to be protective factors, whereas the presence of a duck farm with significant mortality or drop in egg production in the village was found to be a risk factor.

**Conclusions:**

Results demonstrate the key role of several prevention and biosecurity measures to reduce HPAI A (H5N1) virus circulation, which could promote better poultry farm biosecurity in Egypt.

**Electronic supplementary material:**

The online version of this article (10.1186/s12917-018-1519-8) contains supplementary material, which is available to authorized users.

## Background

The highly pathogenic avian influenza (HPAI) A (H5N1) virus has been enzootic in Egyptian poultry for about ten years [1, 2]. Despite years of efforts to control and prevent HPAI A (H5N1) outbreaks, including surveillance, vaccination and culling, the virus continues to spread in the poultry production and trade systems. All along the value chain, from the producers to the end consumers, the risk of exposure of Egyptian farmers to infected poultry is persistent, resulting in sporadic human cases [3–5], some of which lead to severe illness-complications and death (34% case-fatality rate for the severe human cases in Egypt from 2006 to 2015 [5]). The winter of 2014/2015 was particularly significant as the number of human cases in Egypt reached levels never observed before, with 165 new human cases leading to 51 fatalities [4]. This period also marked the emergence of a new cluster of HPAI A (H5N1) virus within the clade 2.2.1.2 [6]. In 2016, the situation more or less returned to the previous levels, with only 10 human cases reported [7].

After nearly 10 years of continued evolution of the HPAI A (H5N1) virus, several distinct antigenic clusters have emerged in Egypt within the clade 2.2, notably the clades 2.2.1, 2.2.1.1 and 2.2.1.2 [1]. The diversity of viral HA gene found in Egypt [1, 5, 8, 9] may be partially explained by the wide range of poultry production systems coexisting in the Nile river delta, ranging from backyard and household ownership to large commercial and industrial farms. Each system is associated with specific mixes of domestic species, different levels of biosecurity, differences in vaccine coverage, different production cycles and value chains [10] resulting in a wide diversity of selection pressures for the HPAI A (H5N1) virus. Although gallinaceous species dominate the commercial and industrial meat and egg production, ducks, geese, turkeys, pigeons and quails are also intensively reared and are very essential and palatable sources of meat in Egypt. The survival, transmission rate and susceptibility for HPAI A (H5N1) infection varies considerably between poultry species, with notably ducks showing less mortality and clinical signs of infection than chickens, turkeys and geese for the clade 2.2.1.2 circulating in 2014/2015 [11]. In such a context, avian influenza (AI) outbreaks in Egyptian poultry industry are difficult to control and economically disruptive on account of direct losses to poultry stock and indirect costs due to the introduction of biosecurity measures in farms. More importantly, they impact the food security of the country.

A comprehensive review of risk factors of HPAI A (H5N1) at different levels (farm, pixel, administrative unit) in countries other than Egypt [12] found duck population density, indicators of human population density or activity, and indicators of water presence or abundance as being the factors most consistently found across studies and scale. At the farm level, the risk or protective factors of HPAI A (H5N1) infection have rarely been assessed with statistical models in Egypt. The high under reporting rate of HPAI A (H5N1) outbreaks due to the lack of compensation measures for poultry lost [13, 14] and/or indirect effects of vaccination campaigns [15], made it difficult to get reliable data. In household and backyard poultry production, the improper disposal of dead poultry and poultry faeces [16] creates lapses in biosecurity. In addition the limited understanding and poor biosecurity procedures during vaccination of household poultry by vaccine injectors [13] are also known to be risk factors of HPAI A (H5N1) infections. A risk assessment of HPAI A (H5N1) virus in Egypt [10] concluded that for commercial farms, the risk associated with movement of people is high and probably the most important mode of transmission of AI between farms. External injectors, part-time day farm workers, visiting veterinary practitioners, feed delivery, egg-collecting and litter collecting drivers - there are many transmission pathways for AI virus introduction and spread in the commercial farms. These results were confirmed by the study of spatial patterns of genetic relatedness of HPAI A (H5N1) in Egyptian poultry from 2006 to 2013 [17]. Genetic information suggests that wild birds play an important role in viral diffusion between backyard farms, while commercial farms experience human-mediated diffusion via the road network. However, the contacts between external visitors and poultry cannot alone explain the spread of HPAI A (H5N1) between farms. The role of biosecurity practices should also be taken into account as these are able to mediate the virus circulation in poultry production systems. Many authors have investigated risk factors at the farm level and reported significant factors linked to the biosecurity categorized into isolation, sanitation and traffic control [18] in the review of the literature presented in Table 1. This review shows that a set of biosecurity risks and biosecurity practices should be considered to estimate the overall level of biosecurity of poultry farms.

**Table 1:** Literature review of biosecurity risks and biosecurity practises against HPAI A (H5N1) virus assessed at farm level

Biosecurity practices Positive impacts	
	Isolation
	• Chicken and ducks in different shelters at night [31]
	Traffic control
	• Designated vehicle for sending eggs to a vendor or market [32]
	• Not allowing traders to enter the farm [33]
	Sanitation
	• Owners used a disinfectant to clean poultry areas [34]
	• Farm staff washing their hands before handling birds [33]
Biosecurity risks	
	Isolation
	• Farm accessible to feral and wild animals [32]
	• Footbath at entry to farm/shed [32]
	• Dead crow seen at or near farm [35]
	• Improperly dispose farm waste [36]
	• Having contact with pigeons [31]
	Traffic control
	• Family and friends visiting [24]
	• Exchanging eggtrays with market vendors [35]
	• Receiving visitors on farm premises [37]
	• Purchased live poultry/products [37]
	• Farm workers live outside the premises [37]
	• Owners bought live chickens from another backyard farm [34]
	• Having a neighbouring poultry farm [33]
	• High frequency of veterinary visits [38]
	• Had visitors in their farm within the past month [36]

The aims of this study were to analyse the risk of HPAI A (H5N1) presence at the farm level in a large part of the Nile Delta, with a particular emphasis on simple factors such as the type of poultry production system, vaccination status, closed vs. open status of the farm, farm size and the presence or absence of duck farms reporting abnormal mortality or drop in egg production in the village in which the farms were located.

## Methods

### Case definition

The study was based on passive surveillance data recorded from 2014 to 2015 in commercial poultry farms in the Western-side of Nile delta in Egypt. Suspected premises were visited following reporting of sudden and high mortality in broiler or duck flocks, or of a drop of egg production in layers or breeders. During investigation of the suspected cases, 3–5 pooled samples from sick or freshly dead birds were tested for HPAI A (H5N1) virus by laboratory tests and confirmed by reverse transcription polymerase chain reaction (RT-PCR). The samples which consisted of a set tracheal and cloacal swabs and organs from trachea, lung and kidneys were processed for virus isolation according to the OIE manual [19]. Each farm was categorized as ‘infected’ if the presence of the HPAI A (H5N1) was confirmed, and as ‘non-infected’ otherwise. All surveyed farms had therefore reported abnormal mortality or a drop in egg production.

### Study area and disease data collection

**Fig. 1:**
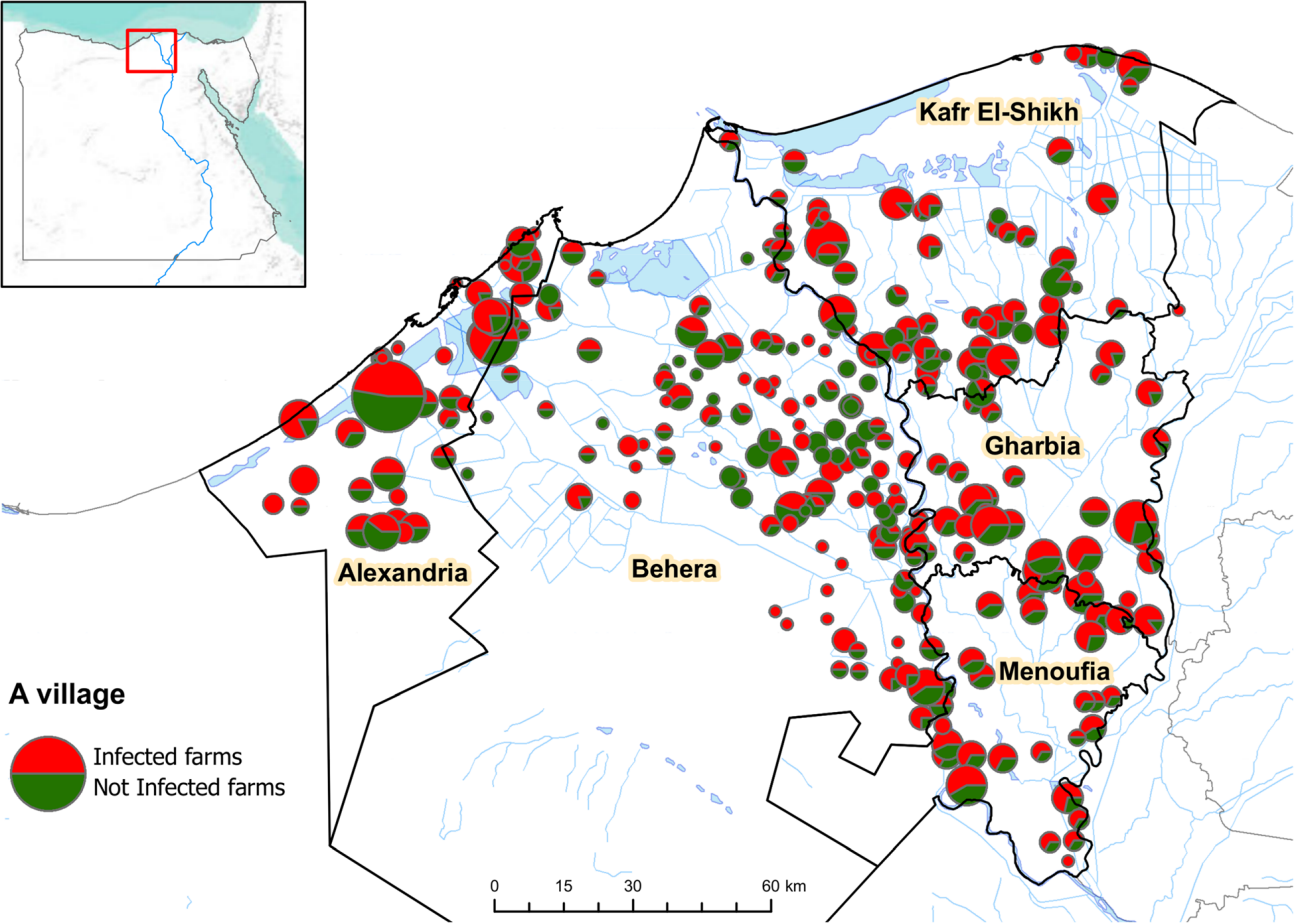
Map of study area (Nile river delta, Egypt), location of farms (the coordinates refers to villages) and village prevalence to HPAI A (H5N1) virus. The size of the circle depends on the number of farms in the village and the colour represents the proportion of positive (red) and negative (green) farms for Avian Influenza in the years 2014–2015

The study area is located on the Western-side of Nile delta, on the Mediterranean coast of Egypt, ranging from 29°25′ to 31°20′ East, 30°12′ to 31°36’ North. The samples were collected from 2014 to 2015 in five governorates (regions), namely Alexandria, Behera, Kafr El-Shikh, Gharbia, Menoufia (Fig. 1). Both chicken (*n* = 746) and duck (*n* = 364) farms were visited and the following information were collected at the time of the visit: species (chicken or duck), farm type (broiler chickens, layer chickens, laying ducks, meat ducks), location (name of the village), date of report, number of animals in the farm, average age and weight of the birds, number of dead birds at the time of the visit, preventive measures implemented in the farm (open vs. closed farm, vaccinated vs. not vaccinated). In an open farm system, windows for the purpose of ventilation are present in the farm structure. Such open farm systems are generally lacking in rodent and peri-domestic bird control. In the closed farm system, ventilation is provided by electric fans or pad cooling systems. The advantage of such a system is improved biosecurity in farms.

With regard to vaccination, presently inactivated vaccines are being used for the control of H5N1, H5N2 or H5N3. In vaccinated flocks, all birds were vaccinated and the recommendations for time of vaccination were provided to the poultry farmers based on the advice of the vaccine manufacturer and Egyptian veterinarian services (Damanhour University, Egypt): i) broiler chickens: day 5–10 (first round); ii) layer chickens: day 10, 40 and 110; iii) meat ducks: day 5, 10; and iv) layer ducks: day 10, 50 and 100. The number of booster doses and the vaccine strain were not available for our dataset. For the geographical coordinates of the farms included in this study, we used the village centroids for protection of privacy.

### Statistical analyses

In order to test the effect of a list of independent variables on the probability of presence of HPAI A (H5N1) virus at farm level (*n* = 1110 farms), a binomial generalized linear model (GLM) was formulated. Initially investigated variables were the type of poultry production (egg or meat) and species (chicken or duck) information, which were combined into a single variable describing the production type (“Ptype”: broiler chicken farms, layer chicken farms, meat duck farms, laying duck farms). The other independent variables included the presence or absence of vaccination for influenza A (H5Nx) virus (“Vaccination”), the closed vs. open farm status (“Closure”), the number of birds per production cycle in the farm (“Nb_Birds”), the presence (1) or absence (0) of at least one visited duck farm in the village (hence reporting abnormal mortality or drop in egg production) in which the farm was located (“HasDuck”). A multivariate GLM binomial model was developed using all individual risk factors. In addition, the bivariate interactions between production type and vaccination, production type and closure status, and production type and the number of birds were tested. The average age and weight of birds of the flocks were not tested because the largest part of the variability in age and weight was confounded by the poultry type variable. We quantified the effects of all simple and interaction terms from a full model (including all the variables interactions) using an analysis of deviance upon removal and a chi-squared test with type II sum of squares procedure. Insignificant variables and interactions were then taken out according to the Akaike’s information criteria (AIC) to derive the final most parsimonious model with a stepwise selection procedure. The final model was also used to visualise the role of each explanatory variable on the prediction, having adjusted for other variables presented in model [20]. The predictive accuracy of the final model was assessed using the Receiver Operating Characteristic curve (ROC). The area under the ROC curve (AUC), sensitivity and specificity indices were computed using a cross-validation (CV) method using 70% of the data to fit the model and the remaining 30% of the data for external validation. A stratified random sampling of the dataset into training and testing sets was used to preserve the virus prevalence inside each subsample during the CV procedure. Finally, the AUC was bootstrapped with 50 different data splits. The presence of spatial autocorrelation in the model residuals was evaluated visually using maps, and quantitatively using spline correlograms [21] and Moran’s I statistics [22].

## Results

A total number of 1110 farms were visited, based on passive reporting of suspected HPAI A (H5N1) in the years 2014–2015 in 5 Egyptian provinces in the delta of Nile River (Fig. 1): 746 chicken farms (501 broiler farms and 245 layer farms) and 364 duck farms (192 meat farms and 172 laying farms). The proportion of HPAI A (H5N1) positive farms was 62.16% (690/1110). This figure was nearly similar for chicken (62.33%, 465/746) and duck (61.81%, 225/364) farms. However, there were notable differences in the proportion of HPAI A (H5N1) positive farms between the poultry types, with 64.87% broiler chicken farms, 57.14% layer chicken farms, 58.33% meat duck farms, and 65.70% laying duck farms being diagnosed as positive. A map of the number of farms per village with the village-level proportion of positive farms can be seen in Fig. 1, highlighting the widespread distribution of HPAI A (H5N1) positive farms in the Nile Delta and the strong spatial heterogeneity of the proportion of positive farms at the village level.

The proportion of farms vaccinated against HPAI A (H5Nx) virus was about 42.43% (471/1110): 43.91% (220/501) for the broiler chicken farms, 48.57% (119/245) for the layer chicken farms, 40.63% (78/192) for the meat duck farms and 31.40% (54/172) for the laying duck farms. The proportion of closed farms was 44.05%: 50.10% (251/501) for broiler chicken farms, 38.37% (94/245) for layer chicken farms, 43.75% (84/192) for meat duck farms and 34.88% (60/172) for laying duck farms.

**Fig. 2:**
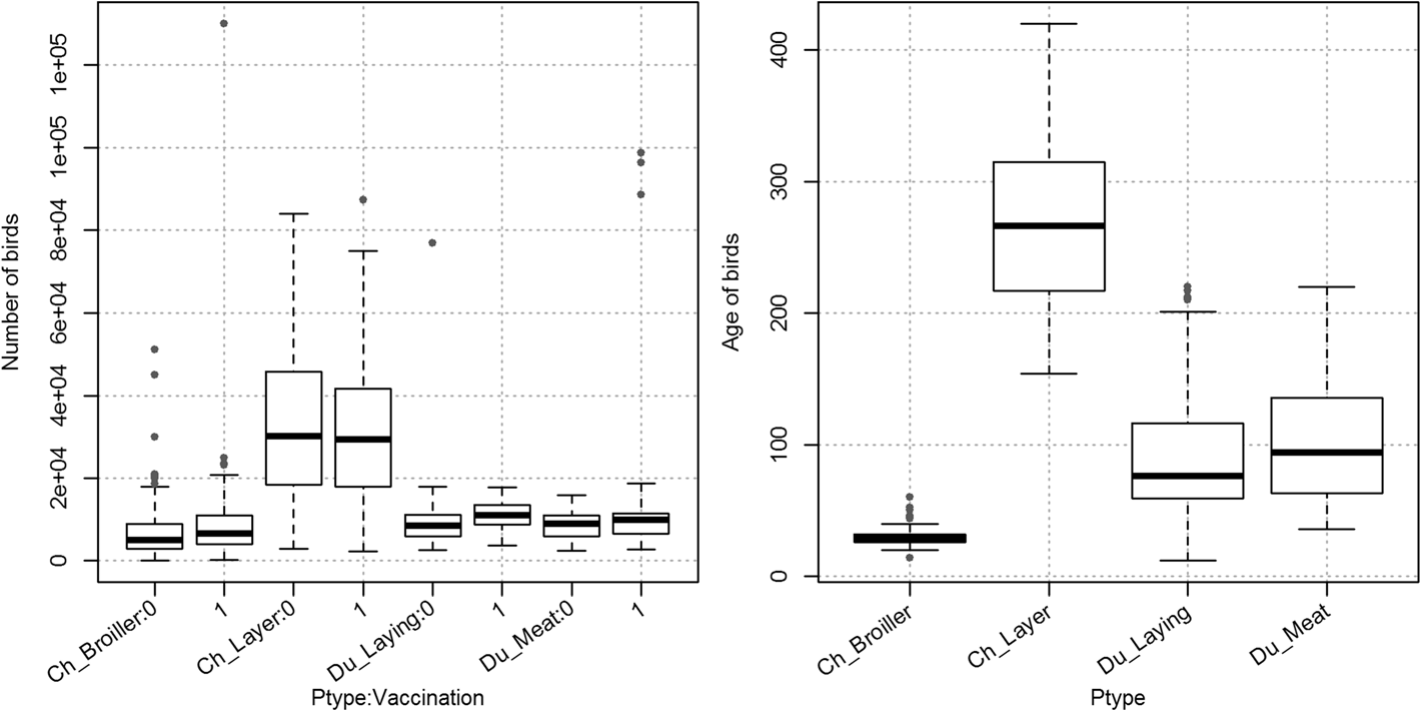
Distribution at farm level of number of birds per cycle (left) and the mean ages of birds (right). The number of birds is split into 8 categories according to the presence (1) or the absence (0) of vaccination in the farms and the four types of the poultry production. The mean age of birds is divided into the four categories of the poultry production

The mean number of birds per farm ranged from 101 to 1.3 × 10^5^ in broiler chicken farms, from 2349 to 8.7 × 10^4^ in layer chicken, from 2401 to 9.9 × 10^4^ in meat duck farms and from 2588 to 7.7 × 10^4^ in laying duck farms. Figure 2 shows the distribution of bird numbers per farm type according to their vaccination status. The median number of birds per farm was higher in layer chicken farms compared to other type of poultry production. One can also note that except for the layer chicken farm category, the median number of birds per farm appeared to be slightly higher in vaccinated farms. Figure 2 also shows the distribution of the mean age of birds per farm type at the time of the visit. Large differences in the mean ages of birds can be noted between the poultry production types.

**Fig. 3:**
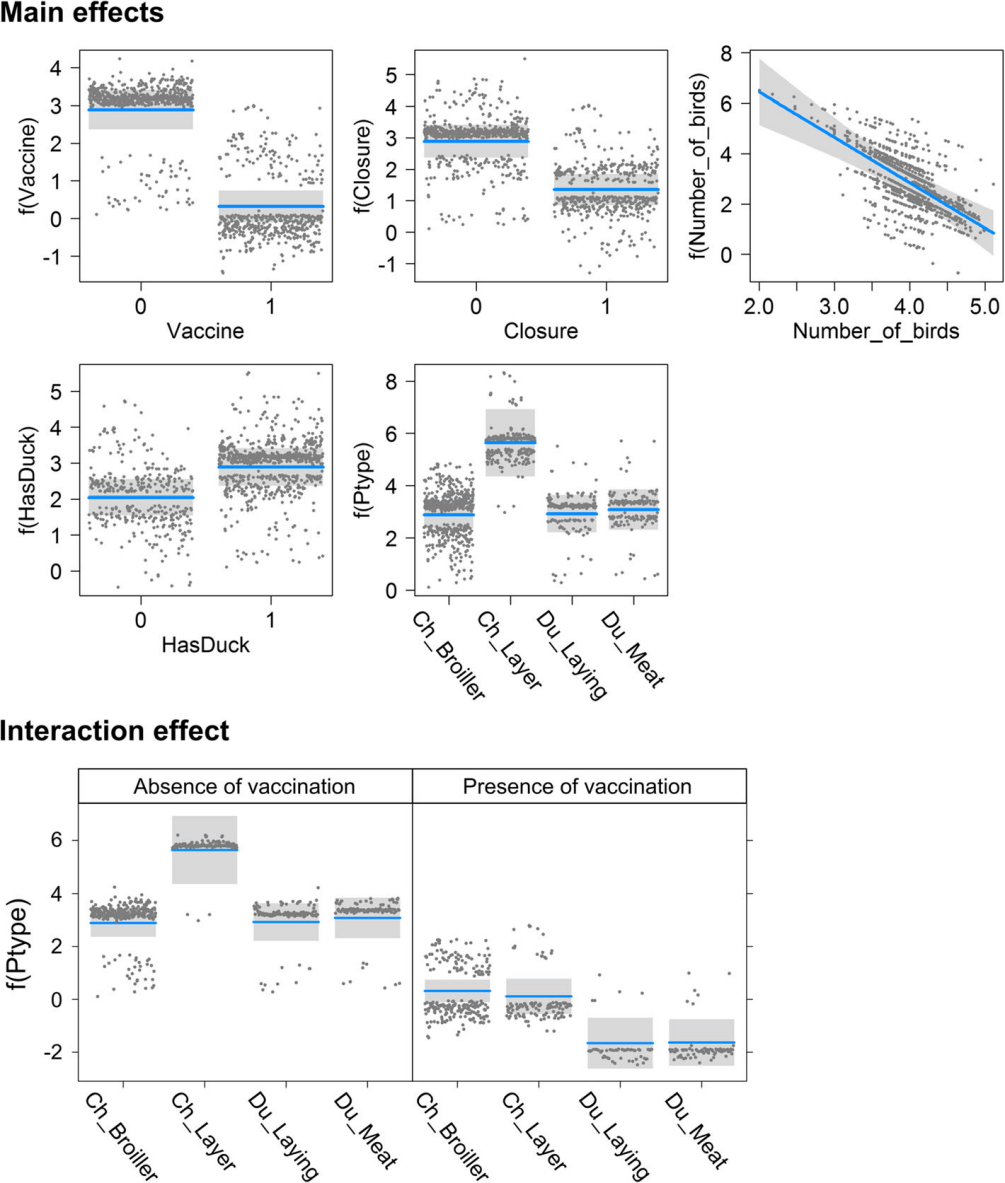
Conditional plots between the probability of presence of AI A (H5N1) virus and the independent variables of GLM (top) or the type of poultry production according to the presence or the absence of vaccination (bottom). Shading represents 95% confidence intervals and points are partial residuals

The goodness of fit of GLM model was high with a mean AUC value of 0.93 and the model residuals showed no evidence of spatial autocorrelation (see Additional files 1 & 2). The analysis of deviance of GLM model run with all the variables and interactions is presented in Table 2, while the estimates of the final model coefficients with the odds ratios (OR) is presented in Table 3. The GLM model found significant differences between the probability of presence of HPAI A (H5N1) virus according to the vaccination status and this probability increases in farms without vaccination. The strongest change in deviance was found for the vaccination status variable (Table 2). This factor was followed by the closure status, the number of birds (the farm size), and the presence/absence of duck farms reporting abnormal mortality or egg losses in the village. The only significant interaction term was the interaction between poultry farm type and vaccination status, indicating that vaccination may affect the probability of HPAI A (H5N1) presence differently in the different farm types. The single term revealed that vaccination status (OR: 0.08, CI: 0.05–0.13), farm closure (OR: 0.22, CI: 0.14–0.32) and bird numbers (OR: 0.17, CI: 0.09–0.30) were protective factors, whereas the presence of duck farms reporting abnormal mortality or egg losses in the village was a risk factor (OR: 2.31, CI: 1.49–3.64). Figure 3 illustrates these relationships, together with the effect of the poultry farm type on the predicted probability of HPAI A (H5N1) presence. The probability of presence of HPAI A (H5N1) virus was significantly higher in chicken layer farms (OR: 15.62, CI: 4.75–71.69) compared to other farm types. Interestingly, this pattern changed in vaccinated farms and the interactions between the types of production variable against the vaccination variable were important in explaining the response variable (Fig. 3). In vaccinated farms, the probability of farm infection is higher in chicken (broiler and layer) farms compared to the duck farms (meat and laying).

**Table 2:** Analysis of deviance from the logistic regression and chi-squared tests

	Deviance	Df	*P*-values
Vaccination	453,326	1	< 0,001
Closure	61,767	1	< 0,001
Nb_Birds	36,159	1	< 0,001
Ptype	28,788	3	< 0,001
HasDuck	11,971	1	0,001
Vaccination:Ptype	29,608	3	< 0,001
Nb_Birds:Ptype	3410	3	0,33
Closure:Ptype	1638	3	0,65

**Table 3:** Estimation of model coefficients and odds ratios from the logistic regression with 95% credible intervals

	Estimate	Std. Error	Odds ratio	2.5%	97.5%
(Intercept)	9,22	1,24	–	–	–
Vaccine	−2,57	0,26	0,08	0,05	0,13
Closure	-1,53	0,2	0,22	0,14	0,32
Nb_Birds	-1,8	0,32	0,17	0,09	0,3
HasDuck	0,84	0,23	2,31	1,49	3,64
Ptype - Ch_Layer	2,75	0,67	15,62	4,75	71,69
Ptype - Du_Laying	0,04	0,42	1,04	0,46	2,47
Ptype - Du_Meat	0,19	0,45	1,21	0,52	3,08
[Vaccine:Ptype] - 1:Ch_Layer	−2,95	0,71	0,05	0,01	0,18
[Vaccine:Ptype] - 1:Du_Laying	−2,01	0,65	0,13	0,03	0,45
[Vaccine:Ptype] - 1:Du_Meat	-2,14	0,63	0,12	0,03	0,38

## Discussion

The results show that the type of poultry production, HPAI vaccination status and the biosecurity level of farms have a strong influence on the presence or absence of HPAI A (H5N1) virus at poultry farm level. In this study, the biosecurity level of farms was estimated through a list of biosecurity practices and biosecurity risks: the absence of windows/openings in farm, the number of birds per cycle of production (practices) and the presence of duck farms in the village (risk). Every variable tested in the GLM model were found significant to explain the probability of HPAI A (H5N1) presence and the discriminatory capacity of model was good with AUC about 0.93. It should be recalled that the model is based only on five independent variables. Figure 1 shows no clear spatial pattern in the distribution of the HPAI A (H5N1) virus among farms and the virus was detected in nearly every village.

Vaccination for HPAI was associated with a decrease in the probability of presence of HPAI A (H5N1) virus and can explain the high goodness of fit of the GLM model. Indeed, the virus was detected in 22.08% (104/471) of vaccinated farm while 91.71% (586/639) of unvaccinated farms were infected showing the good discriminatory capacity of this factor. However, in a few cases (104/471) vaccination failed to prevent the virus from being introduced into the farm. The absence of information about the number of booster doses and the vaccine strain used creates difficulties in interpreting this result. Vaccination in poultry using a non-matching virus vaccine strain to the circulating strain and lack of booster doses have been shown to possibly reduce clinical disease signs, without reducing the effect on virus transmission [23, 24]. Although the cost-benefits of vaccination seem positive at individual farm level, the virus is still persisting in Egypt. The residual circulation of virus in vaccinated farm is an obstacle to prevent the HPAI A (H5N1) virus spreading in Nile river delta.

Then, the risk of HPAI A (H5N1) infection was higher in farms with windows/openings than farms without such types of ventilation in GLM models. This result may suggest that a strict isolation of poultry from wildlife is needed to prevent the farms from infection by the HPAI A (H5N1) virus. Indeed, bridging host species may exist in the peri-domestic populations (terrestrial birds, crows, doves, pigeon, egrets and rodents) frequently found around the poultry farms and some are susceptible to HPAI A (H5N1) [25]. In Egypt, crows, cattle egret and pigeons are naturally infected by H5 viruses [26]. Bridge hosts provide a link through which pathogens can be transmitted from maintenance host populations (from farms and/or wildlife) to receptive populations (the commercial farms) [27]. A FAO report has already pointed the importance [10] of regularly checking and repairing broken windows and the wire screening on the sides of the poultry house to prevent wild bird access to the farms. Moreover, farms without an efficient or modern ventilation system may be an indicator of lack of other investments in the farm infrastructure resulting in overall lower levels of biosecurity.

In the study area 97.57% of studied farms had less than 20,000 birds per cycle with the exception of layer chicken farms, (Fig. 2). Rather, the layer chicken farm production is more intensive and 69.80% of layer chicken farms have more than 20,000 birds per cycle. The results of model showed that the risk of HPAI A (H5N1) infection decreases significantly with the number of birds per cycle of production. The number of birds per cycle provides a figure for estimating the farm size and is a good surrogate of various aspects of the biosecurity level of farms. For example, management practices of farmers can change according to the size and the level of intensification of the poultry production of farms [28]. In this study of 267 broiler chicken farms in the Gharbia Governorate, the farms with more than 20,000 birds per cycle use notably more automatic feeders/waters and assess more systematically the health status of birds before selling, than the farms of more moderate size. Moreover, it was reported in the study by Eltholth et al. (2016) that the farmers of such large farms do not visit other farms whereas they are more inclined to do so in farms of a more moderate size.

The probability of presence of the HPAI A (H5N1) virus in poultry is dependant of the type of poultry production. The GLM predicted that the probability of virus presence was higher for the layer chicken farms while the observed prevalence for this category was lowest (57.14%) compared to other poultry type. The observed virus prevalence is the result of a sum of multiple factors and cannot be directly interpreted. A multiple regression model was needed to separate the effects affecting the probability of farm infection. For example, layer chicken farms have a higher number of poultry per production cycle in average, and the effects associated with this factor can be predominant compared to some other effects linked to the type of poultry production. The higher probability of HPAI A (H5N1) virus in layer chicken farms may be linked to the higher mean age of birds (Fig. 2) in this production system (the age is not taken into account in the model).

The presence of a duck farm reporting abnormal mortality or a drop in egg production in the village where the farm is located was associated with a higher probability of presence of the HPAI A (H5N1) virus in the GLM. In Egypt, duck production is mainly in small commercial or backyard farms with relatively low biosecurity levels. Ducks have long been considered an important risk factor for HPAI A (H5N1) virus presence, and show various levels of clinical signs at the individual level, ranging from high mortality to the absence of clinical signs accompanied with virus shedding [29]. Recent experimental infection of poultry with a HPAI A (H5N1) contemporary virus strain circulating in 2014/2015 [11] shows that unvaccinated ducks showed no symptoms of infection and survived the duration of the experiment. However, this is only theorised here as the authors of this study didn’t test the effect of infection on egg production. This recent virus strain may cause much fewer clinical signs of infection in ducks, allowing virus persistence and spread to other poultry farms, a risk that has already been previously identified [30]. In the specific context of this study, silent circulation in duck farms in the absence of clinical signs could not be formally assessed, because farms reporting no clinical signs were simply not surveyed and identified in the villages. However, given the variability in clinical signs, and the overall high prevalence of HPAI H5N1 in the area, one could reasonably assume that a number of farms with silent circulation could have represented an equivalent, if not greater, risk of transmission to their neighbourhood as those reporting abnormal mortalities or drops in egg production.

The study analysed data from passive surveillance and all surveyed farms had therefore reported abnormal mortality or drop in egg production. So, the results this study should be considered in that specific context, which entails a number of limitations. First, the virus was detected in 62.16% of the tested farms and this figure can therefore not be compared to prevalence figures obtained through cross-sectional surveys that consider all farms. Two types of farms may not have reported abnormal mortality or drops in egg production, for entirely different reasons. On the one hand, some farms with very high biosecurity and thorough implementation of vaccination may never report clinical signs. On the other hand, farms with domestic ducks or specific chicken breeds that have lower susceptibility may also not present clinical signs in birds, and would not be included. So, depending on the relative abundance of those two situations, our apparent farm-level prevalence of 62.16% may be an overestimation or an underestimation of the true prevalence. For the same reason, our measure of the benefits of vaccination at the farm level could be underestimated if many vaccinated farms never report any clinical signs to be included in the survey, or/and were not infected at all, or, conversely, overestimated, if inefficiently vaccinated farms had silent circulation of the virus. Other factors may be at play in influencing the vaccination coverage. For example, in parts of the Nile Delta other than the survey area that experienced fewer recent HPAI A (H5N1) outbreaks, farmers may have a lower perception of the usefulness of vaccination, which could result in lower vaccination coverage.

## Conclusions

The analysis focuses on only a relevant but limited subset of protective or risk factors at the farm level for different type of poultry production systems. However, many other potentially important factors could be considered, such as, for example, the presence of other pathogens, the number of visits to the farms, the turnover of poultry production, the extent of inward (inputs) and outward (outputs) movements, or the position of the farm in the poultry value-chain network. Future investigations could consider such information to provide a more comprehensive assessment of the local risk of AI transmission in poultry.

## Additional files

Additional file 1: Receiver operating characteristic from the logistic regression computed with the training data (black line) and computed with a cross-validation (CV) method (blurred lines). A stratified random sampling of the dataset into training and test sets was used for the CV and the AUC was bootstrapped with 50 different data splitting. (PNG 84 kb)

Additional file 2: The results used in assessing spatial autocorrelation. A) Map of residual deviances of GLM (top): the dot sizes are proportional to the deviance value of each farm. The red dots represent negative residual deviances while the blue dots are the positive residual deviances. B) Correlogram of model residuals (bottom-left). C) Moran’s test for spatial autocorrelation using a spatial weights matrix based on the neighbourhood relations showed in the figure (bottom-right). Two farms were linked together if the distance between these two farms was less than 0.08°. (PNG 202 kb)

## Abbreviations

AI: Avian influenza
AIC: Akaike’s information criteria
AUC: Area under the receiver operating characteristic curve
Closure: Predictor variable related to the closed vs. opened status of the farm
CV: Cross-validation
GLM: Generalized linear model
HasDuck: Predictor variable related to the presence or absence of at least one duck farm reporting abnormal mortality or drop in egg production in the village in which the farm was located
HPAI: Highly pathogenic avian influenza
Nb_Birds: Predictor variable related to the number of birds per production cycle in the farm
OR: Odds ratio
Ptype: Predictor variable related to the type of poultry production
ROC: Receiver operating characteristic curve
RT-PCR: Reverse transcription polymerase chain reaction
Vaccination: Predictor variable related to the presence or absence of vaccination for influenza A (H5Nx) virus
