# Supplementary figures and images for: Avian influenza A (H5N1) outbreaks in different poultry farm types in Egypt: the effect of vaccination, closing status and farm size
Source: BMC Vet Res. 2018 Jun 18;14:187. doi: 10.1186/s12917-018-1519-8 (PMC6006767; doi:10.1186/s12917-018-1519-8)

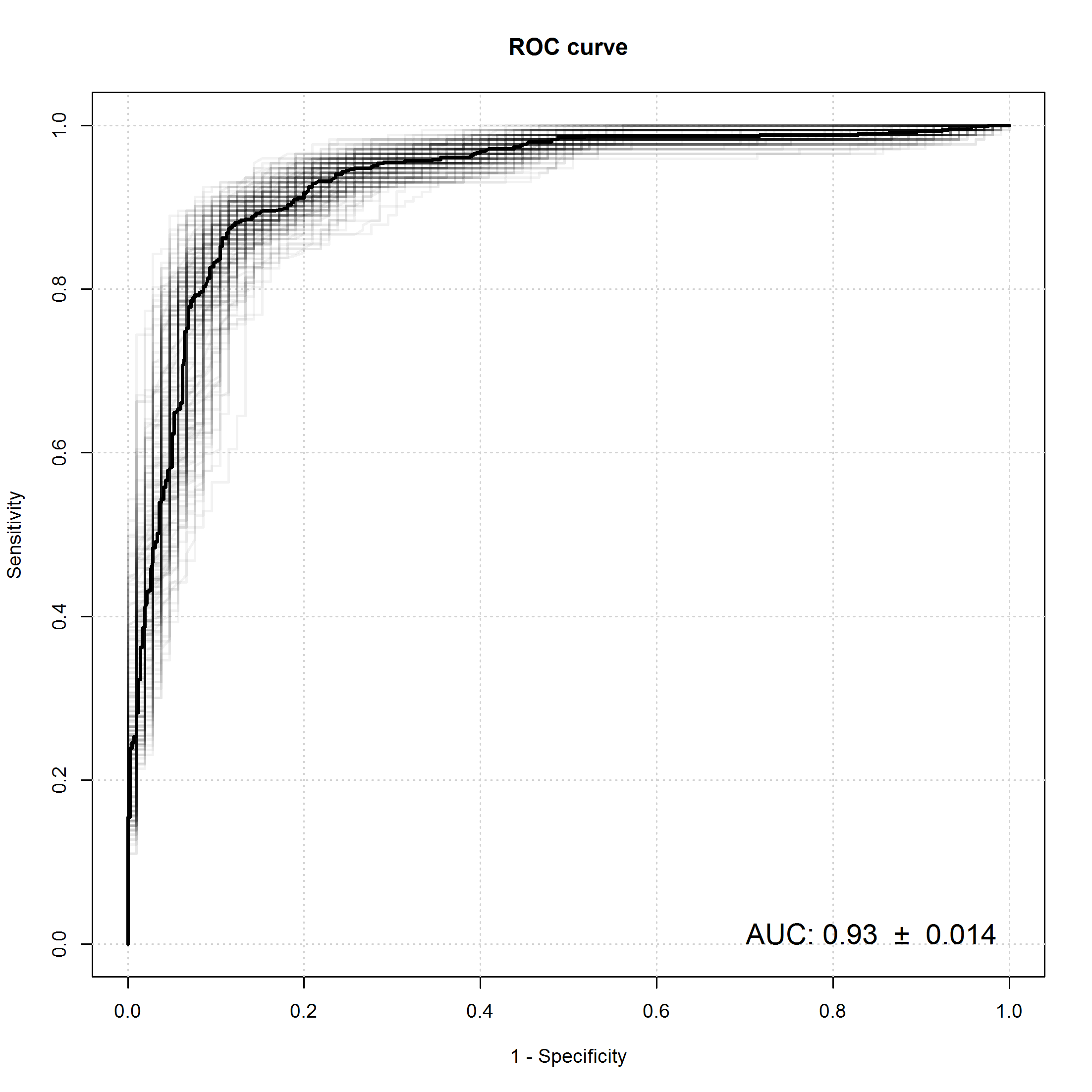

Supplement: Supplementary file 1 — Receiver operating characteristic from the logistic regression computed with the training data (black line) and computed with a cross-validation (CV) method (blurred lines). A stratified random sampling of the dataset into training and test sets was used for the CV and the AUC was bootstrapped with 50 different data splitting. (PNG 84 kb) [file 12917_2018_1519_MOESM1_ESM.png]

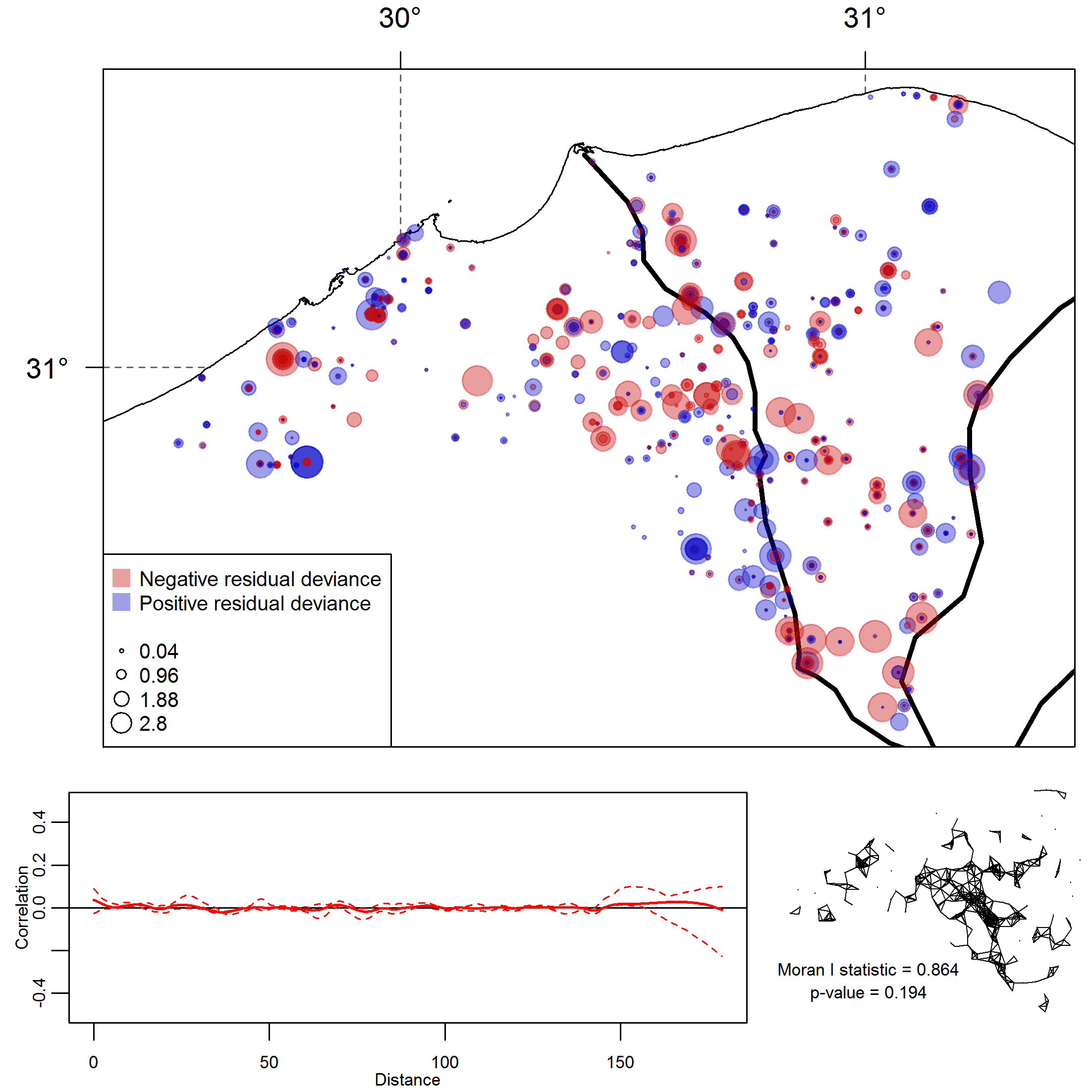

Supplement: Supplementary file 2 — The results used in assessing spatial autocorrelation. A) Map of residual deviances of GLM (top): the dot sizes are proportional to the deviance value of each farm. The red dots represent negative residual deviances while the blue dots are the positive residual deviances. B) Correlogram of model residuals (bottom-left). C) Moran’s test for spatial autocorrelation using a spatial weights matrix based on the neighbourhood relations showed in the figure (bottom-right). Two farms were linked together if the distance between these two farms was less than 0.08°. (PNG 202 kb) [file 12917_2018_1519_MOESM2_ESM.png]
